# A Digital Twin Approach for Contextual Assistance for Surgeons During Surgical Robotics Training

**DOI:** 10.3389/frobt.2021.735566

**Published:** 2021-09-21

**Authors:** Katharina Hagmann, Anja Hellings-Kuß, Julian Klodmann, Rebecca Richter, Freek Stulp, Daniel Leidner

**Affiliations:** German Aerospace Center (DLR), Institute of Robotics and Mechatronics Center, Weßling, Germany

**Keywords:** minimally invasive robotic surgery, shared control, digital twin, virtual fixtures, surgical robotics training

## Abstract

Minimally invasive robotic surgery copes with some disadvantages for the surgeon of minimally invasive surgery while preserving the advantages for the patient. Most commercially available robotic systems are telemanipulated with haptic input devices. The exploitation of the haptics channel, e.g., by means of Virtual Fixtures, would allow for an individualized enhancement of surgical performance with contextual assistance. However, it remains an open field of research as it is non-trivial to estimate the task context itself during a surgery. In contrast, surgical training allows to abstract away from a real operation and thus makes it possible to model the task accurately. The presented approach exploits this fact to parameterize Virtual Fixtures during surgical training, proposing a Shared Control Parametrization Engine that retrieves procedural context information from a Digital Twin. This approach accelerates a proficient use of the robotic system for novice surgeons by augmenting the surgeon’s performance through haptic assistance. With this our aim is to reduce the required skill level and cognitive load of a surgeon performing minimally invasive robotic surgery. A pilot study is performed on the DLR MiroSurge system to evaluate the presented approach. The participants are tasked with two benchmark scenarios of surgical training. The execution of the benchmark scenarios requires basic skills as pick, place and path following. The evaluation of the pilot study shows the promising trend that novel users profit from the haptic augmentation during training of certain tasks.

## 1 Introduction

In the last 30 years, *Minimally Invasive Robotic Surgery (MIRS)* has become an important technology in modern medicine. It leverages the advantages of minimal invasive surgery, such as shorter recovery times for patients, and avoids several of its disadvantages for the surgeons, such as the interrupted hand-eye coordination and the loss of *Degrees of Freedom* (*DoF*) inside the patient’s body (Klodmann et al., 2020). Consequently, MIRS decreases the physical and cognitive load once it has been mastered by the surgeon. However, providing individualized and contextual assistance to enhance surgical performance even further remains an open research topic.

**FIGURE 1:**
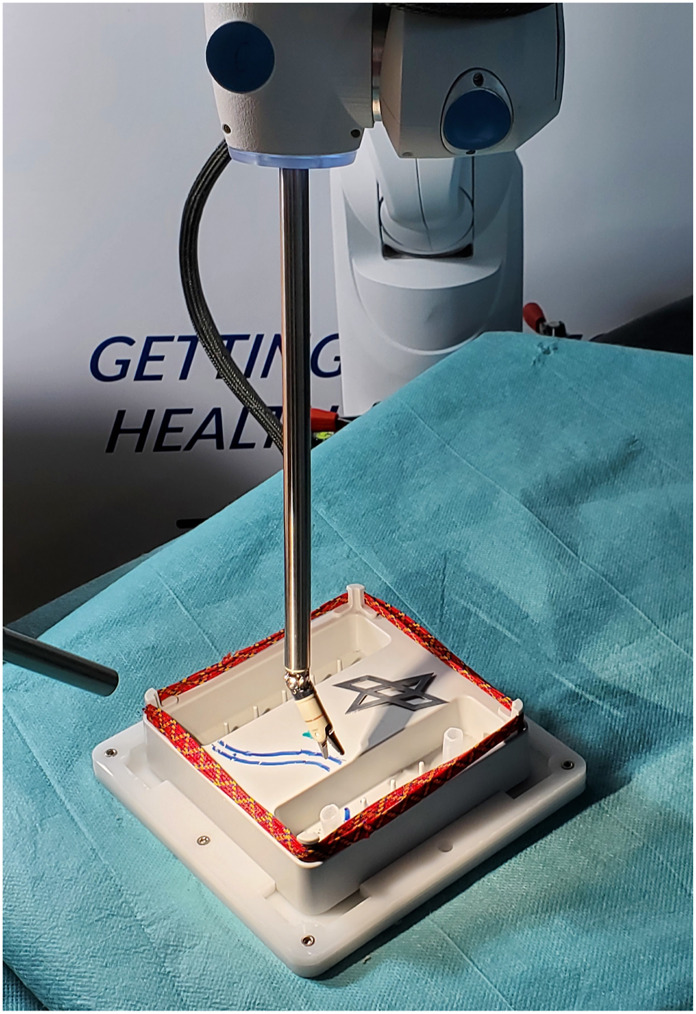
The Nylon Twist training pad from Lübecker Toolbox (LLC, 2021) is performed with the DLR MiroSurge system to train the hand-eye-coordination, bi-manual work, and working over cross.

Exploitation of commonly used haptic input devices, for instance by means of *Virtual Fixtures* (*VFs*), is one possibility in which the surgeon’s performance can be further augmented. However, VFs have to be parameterized according to the context of the task at hand in order to provide reasonable assistance. Due to the unstructured dynamic environment inside the patient’s body, automatic perception of the procedural context in a real surgery remains challenging (van Amsterdam et al., 2021). Surgical training, in turn, offers a well-defined environment. Herein, abstracted training tasks as visualized in Figure 1, are utilized to train key surgical skills.

Our approach introduces a *Digital Twin (DT)* to monitor procedural context information during training. Based on this, we propose a novel concept for the on-line parametrization of haptically rendered assistance functions using VFs. This preserves the dexterity of the surgeon and adds an additional feedback channel to the visual one. We hypothesize that training with our approach enables a novice surgeon to realize a task faster, more accurately and with less cognitive load. The main contributions of this article are 1) an approach to estimate geometric and semantic task states using a DT that mirrors the real robotic system, 2) a novel way to flexibly parameterize assistance functions online based on the estimation of the current semantic task states, 3) the combination of both methods to assist in training for MIRS using multi-modal support, and 4) an evaluation in a proof-of-concept experiment in surgical training.

This work is divided into the following sections. Section 2 further motivates our work based on the current state of the art. The methods used in our approach are explained in Section 3 followed by the description of the pilot study and its results in Section 4. Section 5 presents the conclusions and an outlook.

## 2 State of The Art

Surgical training tasks such as the Lübecker Toolbox (LLC, 2021) offer various training scenarios that are relevant for real operations, as shown in Figure 1. They are designed to train preparation, resection and reconstruction skills. This sequence maps in parts to a cholecystectomy (removal of the gall bladder) as it requires the surgeons to expose critical structures consisting of the cystic duct and the cystic artery by removing surrounding tissue, followed by inserting multiple clips at the correct locations to stop the fluid flow in these structures so they can be cut to free the gall bladder. Even though modern training curricula for MIRS (Thornblade and Fong, 2021) extend traditional surgical training (Sealy, 1999), it is still cumbersome to acquire the required motor skill level to control a surgical robot for the use in MIRS.

Increased autonomy of surgical robots could decrease the required skill level. Haidegger (2019) introduces a scale to classify the level of autonomy in robotic surgery ranging from no autonomy to full autonomy. Commercially available surgical robotic systems assisting in laprascopy fulfill the criteria for level 1 named *robot assistance*, whereas prototypes of surgical robots reach *task-level autonomy* (level 2). Robotic systems in other surgical domains that tackle tasks like autonomously taking blood samples reach up to level 4, *high-level autonomy*. A great challenge of laparascopic surgery consist of the development of perception algorithms, that extract information from the unpredictable environment containing soft tissue. Therefore, autonomous navigation in MIRS is still challenging (Nagy and Haidegger, 2019).

The introduction of assistance functions by means of shared control (Endsley, 1999) offers a possible solution to support surgeon training for MIRS. As a subsequent step the assistance could transfer to real OR situations. Therefore, shared control could be seen as an intermediate step towards the increase to *task-level autonomy*. Assistance functions may range from displaying relevant data using mixed reality (Qian et al., 2019), over tremor filtering and scaling of input movements (Weber et al., 2013) to the automation of certain steps of a task under the supervision of the surgeon (Nagy and Haidegger, 2019). VFs as described in Rosenberg (1993), Abbott et al. (2007), and Bowyer et al. (2014) represent a different type of shared control which enables haptic augmentation. They comprise of attractive VFs that haptically guide the user towards a goal structure as in Selvaggio et al. (2018), repulsive VFs protecting him or her from entering in prohibited zones as in Li et al. (2020), and automated scaling of the user input compared to the instrument tip’s movement as in Zhang et al. (2018). Ovur et al. (2019) show that haptic guidance during training in simulation for simple and complex path-following tasks improves performance. Enayati et al. (2018) assess the specific competences and the overall skill level of novice and expert users during training of a path-following task and adapt the strength of assistance functions according to the competence level.

It is essential that assistance functions are represented such that they can be rapidly adjusted to the ongoing task and the dynamically changing environment. In cholecystectomy for instance, critical structures are first exposed, and it is crucial not to harm them during this step. But in later phases, they are manipulated and even cut. Task-dependent shared control functions are required to account for such drastic change of the task context. The problem of adapting shared-control functions online to a changing environment is stated in Selvaggio et al. (2018) and tackled by an human-in-the-loop approach using VFs. The surgeon can generate attractive VFs and adapt their position and geometry by recording interaction points. Fontanelli et al. (2018) design a vision-free pipeline to support surgeons during suturing. A state machine defines the task. The transitions between states are based on predefined gestures and are, therefore, controlled by the surgeons. Different controllers with varying degree of autonomy and VFs are applied. Maier et al. (2019) develop a training simulator for orthopedic hand surgery in virtual reality generating haptic impressions similar to bone cutting for the surgeon using VFs. A state machine is used to switch between different implementations of VFs according to the task state. All environment states are pre-known due to the virtual reality approach.

Human-in-the-loop approaches for the positioning and parametrization of VF add to the surgeon’s workload. Ideally, the parametrization of VFs should be done automatically by robotic system based on semantic knowledge about the task phases and context, i.e., an abstract knowledge representation, about the environment and the state of the system. A DT approach offers the possibility to derive semantic knowledge from fused data obtained by different sources. A robotic system provides diverse data about its poses and control states. Laaki et al. (2019) design a DT of a simplified surgical environment containing one robotic arm that is controlled in a virtual reality environment by a HTC vive system. The hand movements of the user control the DT in virtual reality and are additionally projected to a robot arm in real-time. Modern machine learning frameworks may eventually be able to segment semantic information of endoscopic images as stated by Scheikl et al. (2020); however, Ahmed and Devoto (2020) consider the modeling of tissue with deformations and movement as the main limitation of DT technologies in surgical robotics. Nevertheless DT-based assistance functions may very well be able to assist in training surgeons where the environment is well-defined by abstracted training pads. In other domains we have shown that physics simulations are able to yield semantic information about the environment as seen in Bauer et al. (2018).

## 3 Methods

**FIGURE 2:**
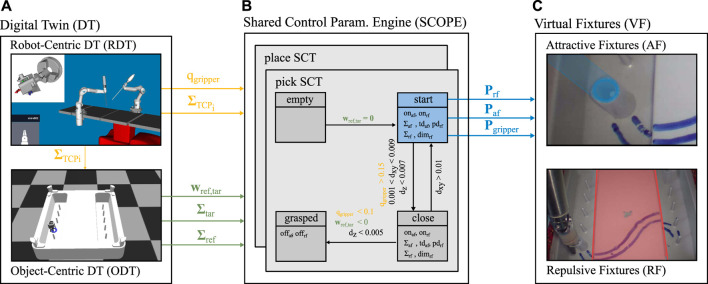
Overview of the three core components and their interaction within the proposed concept. Virtual fixtures **(A)** are introduced in Section 3.1. The parameters are determined by SCOPE **(B)**, which is described in Section 3.2. SCOPE requires the task context, which is provided by the digital twin DT, **(C)**, as described in Section 3.3.

The approach described in this work consists of three modules as visualized in Figure 2. That is, a parameterizable concept for *Virtual Fixtures (VFs)*, the *Shared Control Parametrization Engine (SCOPE)*, and a *Digital Twin (DT)* that provides the required semantic state information. The three modules are introduced in the following and explained on the basis of the benchmark scenarios for in the pilot study. The benchmark scenarios consist of two variations of a pick and place task. They are designed to act on the Nylon Twist training pad as introduced in Figure 1 and are conducted using the DLR MiroSurge surgical robotic platform (Schlenk et al., 2018; Seibold et al., 2018).

For this work one 7-DoF light weight robotic arm called MIRO is mounted on the left standard side rail of the operating room table. The MIRO carries a wristed surgical instrument *i* whose additional 2-DoFs are actuated by a MICA drive unit. The pose of the instrument’s *Tool-Center Point (TCP)* ***Σ***_*TCP*_ is telemanipulated by a Force Dimension’s sigma.7, a 6-DoF input device with an additional gripping DoF, offering the possibility to haptically render forces (Dimension, 2021). A second MIRO is mounted on the right side of the operating room table holding a stereo endoscope. In telemanipulation mode, a software layer integrates multiple avatar robots and maps the motion of up to two input devices to $\Sigma_{TCP_{i}}$ and the endoscope $\Sigma_{TCP_{e}}$ of the position-controlled robotic system. The mapping is based on an inverse kinematics, which preserves the motion constraints at the incision point, called trocar point ***Σ***_*trocar*_.^1^

### 3.1 Virtual Fixtures

In the following paragraphs, some general design principles of VFs for a surgical robotic platform are explained followed by the description of the implemented VFs, which are divided into system related and parameterizable, task related VF.

VF generate a virtual wrench ***w***_*POI*_ = [***f***_*POI*_; ***m***_*POI*_], comprising the forces ***f***_*POI*_ and torques ***m***_*POI*_, which act on the *Pose of Interest* (*POI*) ***Σ***_*POI*_. A virtual proxy ***Σ***_*proxy*_ mirrors ***Σ***_*POI*_. The motion of ***Σ***_*proxy*_ is restricted by the virtual object that describes the geometries of the VF, i.e. ***Σ***_*proxy*_ stays on the surface of the virtual object while ***Σ***_*POI*_ penetrates it. ***Σ***_*proxy*_ is linked to ***Σ***_*POI*_ by a six DoF spring damper system as explained by Caccavale et al. (1999). The displacement between ***Σ***_*POI*_ and ***Σ***_*proxy*_ generates ***w***_*POI*_ depending on the stiffness ***K*** and the damping ***D***. ***D*** is scaled by a function taking into account the distance between ***Σ***_*POI*_ and ***Σ***_*proxy*_ to avoid an abrupt increase of the damping wrench when a displacement occurs.

The resulting ***w***_*POI*_ is rendered to the input device. If $\Sigma_{POI}=\Sigma_{TCP_{i}}$, the wrench ***w***_*POI*_ is scaled and transformed taking into account the hand-eye-coordination matrix (Tobergte, 2010; Steidle et al., 2014). However, VFs applied to the tool body (e.g., the wrist or shaft) or the robot’s joints require transforming the resulting wrenches to $\Sigma_{TCP_{i}}$ before rendering them to the input device. In a minimally invasive surgical setup, these transformations have to preserve the constraints at ***Σ***_*trocar*_. Therefore, an augmented task vector ***v***_*a*_ and the respective augmented Jacobian ***J***_*a*_, combining the 6-DoF task at $\Sigma_{TCP_{i}}$ with the 2-DoF constraint at ***Σ***_*trocar*_ are set up according to Siciliano and Khatib (2008).

Considering an 8-DoF robot (6-DoF robotic arm and a 2-DoF instrument) ***J***_*a*_ is invertible, so an unique mapping between the joint torques ***τ*** and the Cartesian wrench $w_{TCP_{i}}$ exists. Thus, a unique mapping between ***q***_*robot*_ and ***v***_*a*_, as well as between ***τ*** and the Cartesian wrench ***w***_*a*_ exist. ***w***_*a*_ comprises $w_{TCP_{i}}$ and the wrench ***w***_*trocar*_ at ***Σ***_*trocar*_ counteracting the trocar constraints. To map ***w***_*POI*_ to $w_{TCP_{i}}$, it is first transformed to joint space by ***J***_*POI*_ and finally back to the $\Sigma_{TCP_{i}}$ by

$$\left(\begin{array}{c} w_{TCP_{i}}\\ w_{trocar} \end{array}\right)=J_{a}^{-T}\tau =J_{a}^{-T}J_{POI}^{T}w_{POI}.$$ (1)

With this general implementation several VFs can be realized and haptically rendered to the input device.

#### 3.1.1 System Related VF

**FIGURE 3:**
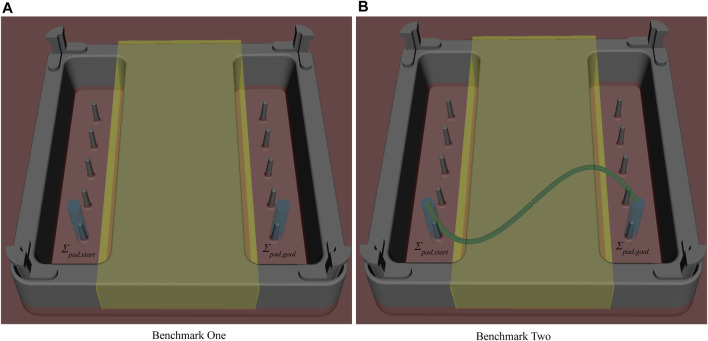
The training pad used for the benchmark scenarios is shown. ***Σ***_*pad*,*start*_ is the initial pose of the ring and ***Σ***_*pad*,*goal*_ its goal pose. The blue cylinders depict the AFs. The yellow box depicts the RF and the red area visualizes the Cartesian workspace limit in *z*-axis. Together they help avoid collisions between the instrument and the trainings pad. **(A)** depicts the first benchmark scenario consisting of an unconstrained pick and place task. **(B)** displays the second benchmark scenario. The constrained pick and place task is realized by an sinus-shaped AF for the follow_path SCT. It is shown by the green curve.

System related VFs are defined and parametrized by the restrictions that come with the robotic system e.g. the length of the instrument’s shaft or workspace limits of the hardware (in Cartesian and Joint space). For the pilot experiments a Cartesian workspace limit on the *x*-axis is set to the bottom of the trainings pad as visualized in Figure 3. According to Nitsch et al. (2012) the intuitiveness is enhanced when haptically guiding the user to avoid such limits by rendering the restrictions to the input devices.

#### 3.1.2 Task Related VF

*Repulsive VFs (RF)* inside the workspace prevent the user from hitting a forbidden region. RFs are implemented using geometrical primitives e.g., virtual boxes as depicted on the right lower side in Figure 2. Their corresponding parameters ***P***_*rf*_ are displayed in Table 1. When ***Σ***_*POI*_ penetrates the virtual box, the ***Σ***_*proxy*_ stays on the surface. Depending on the instrument and the obstacle geometry multiple ***Σ***_*POI*_ can be defined along the instrument structure. For the pick skill RFs in the form of virtual boxes are implemented with $\Sigma_{POI}=\Sigma_{TCP_{i}}$ to avoid crashes between $\Sigma_{TCP_{i}}$ and the training pad.

**TABLE 1:** This table displays the corresponding parameters of RF and AF.

Type	Parameter	Name	Description
***P***_*rf*_	*on*_*rf*_, *off*_*rf*_	Activation	Turns RF on or off
	***Σ***_*rf*_	Pose	Sets middle point of the RF
	*dim*_*rf*_	Edge length	Sets the size of the RF
	*pd*_*rf*_	Max. penetration depth	Sets how far ***Σ***_*POI*_ is allowed to penetrate the RF
***P***_*af*_	*on*_*af*_, *off*_*af*_	Activation	Turns AF on or off
	***Σ***_*af*_	Pose	Sets middle point of the AF
	*td*_*af*_	Translational dead zone	Sets a zone where the AF is not applying forces
	*rd*_*af*_	Rotational dead zone	Sets a zone where the AF is not applying forces
	*s*_*f*_	Force scale	Scale of generated forces
	*s*_*m*_	Torque scale	Scale of generated torques

**FIGURE 4:**
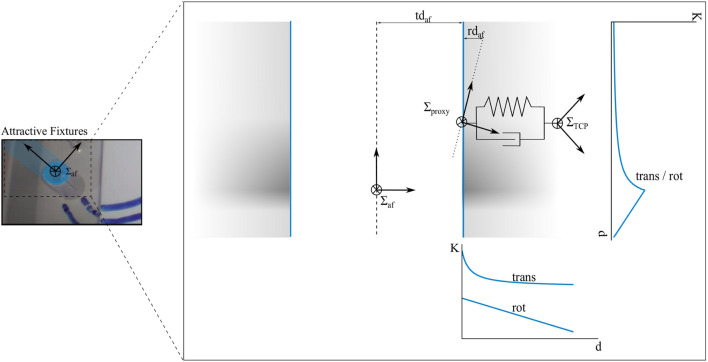
The profile of the cylindrical AF at ***Σ***_*AF*_ is displayed. ***Σ***_TCP_ entered the cylinder, ***Σ***_*proxy*_ stayed at its surface and a virtual spring damper system is spanned in between. The blue lines denote the shape of the stiffness *K* depending on the distance *d* along axial and radial axes of the cylinder. The gray areas depict the blending of the scaling functions.

*Attractive VFs (AF)* guide the user towards or along a certain predefined path or target as visualized on the right upper side in Figure 2. AFs are implemented by a cylindrical geometry around the attractive pose ***Σ***_*af*_, where ***Σ***_*proxy*_ is free to move along the *z*-axis. Along the remaining axes ***Σ***_*proxy*_ is limited by a given radius, which is encoded in the translational dead zone *td*_*af*_. The rotation of ***Σ***_*proxy*_ is fixed to guide the orientation of ***Σ***_*POI*_ with a parameterizable rotational dead zone *rd*_*af*_. Table 1 presents an overview of the parameters of AFs ***P***_*af*_. The pick skill sets ***Σ***_*af*_ to the middle point of a ring with *td*_*af*_ equal to the ring radius to align $\Sigma_{TCP_{i}}$ with the designated grasp pose. *s*_*f*_ and *s*_*m*_, scaling the stiffness, are implemented as function of the distance *d* between ***Σ***_*af*_ and ***Σ***_*POI*_ (see Figure 4). This stiffness function results in an increasing wrench with decreasing *d*. This ensures gentle guidance that the user can easily overcome for large *d* and a strong guidance for small *d*. It was chosen empirically to provide intuitive and subtle guidance to the user.

The second benchmark scenario implements the follow path skill. It restricts the translation from the left side of the trainings pad to the right side by the shape of a polynomial function. The path following AF is implemented by updating ***Σ***_*af*_ in every time step to slide along the predefined path. ***Σ***_*af*_ is defined as the projection of ***Σ***_*POI*_ onto the path with the smallest distance. *s*_*m*_ = 0 is set to let the user control the rotation of ***Σ***_*POI*_.

A virtual wall introduced at the gripping DoF of the input device assists the grasping of objects. The user feels a small opposing force at the point where the gripper of the instrument closes forcelessly. The user overcomes it by pressing slightly against it and the gripper closes firmly applying force on the grasped tissue. The grasp is loosened applying the same principle.

Different phases of a task may require distinct VF. Parameterizable VFs offer an interface to activate them and to adapt their parameters at run time. In the presented work the switching is realized by gradually fading down the initial wrench difference the involved VFs have at the time of switching. Switching between VFs may cause instabilities. While in practice instabilities aren’t observed, a time domain passivity controller as introduced in Hannaford and Ryu (2002) and further developed in Hertkorn et al. (2010), Ryu et al. (2005), and Hulin et al. (2021) is to be integrated into the presented approach in the future, to guarantee a safe operation for arbitrary tasks in all circumstances. Switching between VFs and their parametrization allows the flexible application of various VFs depending on the required task.

### 3.2 Shared Control Parametrization Engine

SCOPE provides a framework to parametrize VFs depending on the current task state. The SCOPE concept builds on our former work on *Shared Control Templates (SCTs)* (Quere et al., 2020). SCTs represent robot skills as a sequence of states leading to a certain goal state as depicted in the center of Figure 2. One SCT may encode different phases in order to guide the user through different phases of a task. In each phase different parameters can be set and different transition conditions apply (e.g., the distance between the end-effector and the target object).

In Quere et al. (2020), SCTs have been implemented to assist in tasks like pick and place, pouring, drinking, or opening doors and drawers. In this work, the portfolio is extended for MIRS tasks required to solve the Nylon Twist training pad from Lübeck Toolbox (see Figure 1), i.e., pick, follow_path, and place. Each of these SCTs describes the interaction between a reference object or a robot component *O*_*ref*_ and a target object *O*_*tar*_ as it is listed in Table 2.

**TABLE 2:** Mapping of *O*_*ref*_ and *O*_*tar*_ for the individual SCTs implemented for MIRS assistance.

	*O*_*ref*_		*O*_*tar*_	
SCT	Type	Pose	Type	Pose
Pick	robot	$\Sigma_{TCP_{i}}$	object	***Σ***_*ring*_
follow_path	robot	$\Sigma_{TCP_{i}}$	object	***Σ***_*pin*_
Place	object	***Σ***_*ring*_	object	***Σ***_*pin*_

SCOPE progresses through the SCTs to parameterize haptically rendered assistance functionality as described in Algorithm 1. Until the goal state is reached SCOPE queries the actual state of the environment and the robot. It receives the current task context C to check if it is possible to progress to the next state. If a state transition is initialized, the new parameters ***P***_*rf*_, ***P***_*af*_, ***P***_*gripper*_ encoded in this state are used to parametrize and activate VFs.

Algorithm 1 Pseudocode of the SCOPE main loop to parse SCTs and parameterize VFs.

**Input:** The *SCT* encoding the current task.

**Output:** The algorithm terminates nominal as all states of the *SCT* are processed.

1 *state*_*id*_ ← 0
2 *state*_*goal*_ ← getGoal (*SCT*)
3 *O*_*ref*_, *O*_*tar*_ ← getReferenceAndTarget (*SCT*)
4 **while** *state*_*id*_ ≠ *state*_*goal*_ **do**
5  C← getContextInformation (*O*_*ref*_, *O*_*tar*_)
6  *state*_*curr*_ ← getCurrentState ($C,SCT,state_{id}$)
7  **if** *state*_*id*_ ≠ *state*_*curr*_ **then**
8   ***P***_*rf*_, ***P***_*af*_, ***P***_*gripper*_ ← getParameter (*state*_*curr*_)
9   parametrizeVF(***P***_*rf*_, ***P***_*af*_, *P*_*gripper*_)
10  *state*_*id*_ ← *state*_*curr*_
11 **return** True

The SCOPE approach relies on current updates of ***Σ***_*ref*_, ***Σ***_*tar*_, ***w***_*ref*,*tar*_, and *q*_*gripper*_ which are included in the task context C provided by the function getContextInformation. A physics-based state inference utilizing the proposed DT model is used to generate the information provided by getContextInformation. This extends the SCT approach which infers the required information through visual perception only.

### 3.3 Digital Twin

The DT approach offers the possibility to generate the required information about the world and the current task and to fuse it with data about the robot obtained by different sources. The DT is divided into a *Robot-centric DT (RDT)* and an *Object-centric DT (ODT)* which constantly exchange information as shown on the left in Figure 2.

The RDT mirrors the DLR Mirosurge system by interfacing its telemanipulation layer. It provides information about the interaction of the different robots. Each MIRO robot generates data at different granularity levels like sensor readings, joint angles and control states. Additionally, information about the relations of the different robots, their workspaces and the poses of their bases are gathered. The RDT offers an interface to select information needed for a task and to provide it for further processing. A middleware provides the infrastructure to query all this information in real time. A viewer displays all relevant information and, therefore, serves as a visualization of the RDT. In Figure 2 the RDT of the DLR MiroSurge system is displayed on the top left. For the pilot study it gathers information about $\Sigma_{TCP_{i}}$ and can acquire further information about the robot.

The ODT provides information about the world state. It makes use of the world state representation as presented in Leidner (2019), which provides an interface to instantiate the robot’s believe about the current state of the world and query this information. It has knowledge about the present objects and their poses. The objects’ poses ***Σ***_*o*_ are dynamically updated. The information about ***Σ***_*o*_ can be provided by an imaging system. However, reasoning about the current state of a task not only requires knowledge about the involved objects and their poses, but also about their interactions. Interactions between objects are encoded in semantic states. E.g. “*ring is grasped by gripper*” requires information about interaction wrenches between the ring and the gripper. In this work we use a physics simulation to keep track of ***Σ***_*o*_ of the objects of interest as it gives us the possibility to generate interaction wrenches between $\Sigma_{TCP_{i}}$ and ***Σ***_*o*_ and derive semantic states.

Pre-known information about a variety of objects is provided by the object database, presented in Leidner (2019), offering a hierarchical structure for objects with respect to their functionality. Physical objects are derived from high level abstract object definitions which provide not only information about the object but also about actions. These can be refined for certain types of the abstract objects. The physics simulation queries the meshes of present objects from the object database and receives their poses from the world state representation. Then it instantiates the objects that are currently present in the robot’s environment. Furthermore, it loads the instrument’s gripper and places it at $\Sigma_{TCP_{i}}$, which is acquired from the RDT and updated in real-time (1 kHz). ***Σ***_*o*_ of all objects can be simulated and is provided to the world state representation. Additionally, this allows the computation of interaction wrenches ***w***_*ref*,*tar*_ between *O*_*ref*_ and *O*_*tar*_, e.g., between the instrument and different objects, and the deduction of semantic states. Figure 2 shows the visualization of the ODT on the bottom left.

The proof-of-concept implementation uses AMBF physics simulation engine (Munawar et al., 2019) which integrates the Bullet Dynamics Engine (Coumans, 2015) as a dynamic solver and interfaces of CHAI-3D (Conti, 2003). This simulation framework was developed to meet the requests of closed-loop kinematic chains and redundant mechanisms as often used in surgical robotics.

This DT approach enables the implementation of the getContextInformation function as explained in Algorithm 2. It decides if *O*_*ref*_ is an object or a robot component and returns the accumulated context information C accordingly.

Algorithm 2 Pseudocode of the getContextInformation function

**Input:** The reference object/robot *O*_*ref*_ and the target object *O*_*tar*_.

**Output:** The algorithm returns context information C from the combined DT state.

1 **if** *O*_*ref*_ is robot **then**
2  ***q***_*gripper*_, ***Σ***_*ref*_ ← getUpdateRDT (*O*_*ref*_)
3  ***Σ***_*tar*_, ***w***_*ref*,*tar*_ ← getUpdateODT (*O*_*tar*_)
4  $C\leftarrow q_{gripper},\Sigma_{ref},\Sigma_{tar},w_{ref,tar}$
5 **else**
6  ***Σ***_*ref*_, ***Σ***_*tar*_, ***w***_*ref*,*tar*_ ← getUpdateODT (*O*_*ref*_, *O*_*tar*_)
7  $C\leftarrow \Sigma_{ref},\Sigma_{tar},w_{ref,tar}$
8 **return** C

## 4 Evaluation

The presented approach is evaluated in a pilot study. The experimental setup is described in subsection 4.1. The results are presented in subsection 4.2.

### 4.1 Experiment Setup

The evaluation is conducted by means of the Nylon Twist training introduced in Figure 1. Two benchmark scenarios are derived as visualized in Figure 4. First, an unconstrained pick and place task ([a]) and second, a constrained pick and place task ([b]). A ring is initially placed on the training pad at the start pose ***Σ***_*pad*,*start*_. The subjects are tasked to pick up this ring and place it on the goal pin located at ***Σ***_*pad*,*goal*_. In benchmark one, the transition between ***Σ***_*pad*,*start*_ and ***Σ***_*pad*,*goal*_ is not constrained as shown in 4 [a]. The benchmark scenario two restricts the transition from ***Σ***_*pad*,*start*_ to ***Σ***_*pad*,*goal*_ to a sine-shaped path as shown in 4 [b]. The predefined path is marked on the real training pad for visual guidance.

The gripper of the instrument is simulated in the ODT at $\Sigma_{TCP_{i}}$. The RDT updates $\Sigma_{TCP_{i}}$ with a rate of 1 kHz. The training pad is located at ***Σ***_*pad*_. In the beginning of each trial, the ring is located at ***Σ***_*ring*_ = ***Σ***_*pad*,*start*_ in the real world and, therefore, also in the ODT. The physical state of the ***Σ***_*ring*_ is simulated by the ODT based on the interaction wrenches between the ring, the environment (i.e., the training pad), and the gripper. Altogether, the DT provides $\Sigma_{TCP_{i}}$ and ***Σ***_*ring*_, the start and goal position of the ring ***Σ***_*pad*,*start*_ and ***Σ***_*pad*,*goal*_, and the gripper angle *q*_*gripper*_. Furthermore, the interaction wrenches $w_{ref,tar}=w_{TCP_{i},ring}$ between $\Sigma_{TCP_{i}}$ and ***Σ***_*ring*_ are generated by the physic simulation.

**FIGURE 5:**
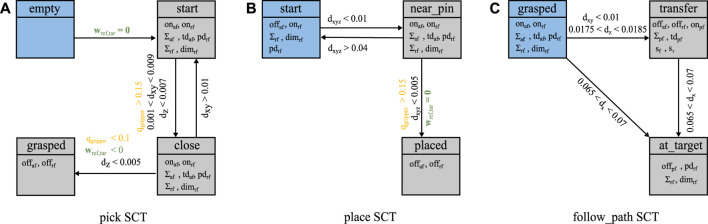
States, transitions and the respective parameters describing the pick SCT **(A)**, the place SCT **(B)**, and the follow_path SCT **(C)**. The blue states indicate the starting states of the respective SCT. The yellow information are provided by the RDT, the green by the ODT and the black are computed as a combination of ODT and RDT.

The proof-of-concept implementation of SCOPE provides three SCTs, namely pick, place, and follow_path as shown in Figure 5. References and targets are visualized in Table 2. The implemented AFs support the successful completion of the task. The Cartesian workspace limit and the RF prevent collisions between the instrument and the training pad. The pick SCT is visualized in Figure 5A with the transition requirements and the corresponding parameters. The state init is the entry point of the pick SCT. If there is no wrench $w_{TCP_{i}}$, the gripper is empty and the transition to state start is performed. The transition to state close is constrained by the distance *d*, defined between $\Sigma_{TCP_{i}}$ and $\Sigma_{TCP_{ring}}$, and *q*_*gripper*_. The state grasped is reached if *d*_*z*_, the distance along the *z*-axis, is sufficiently small, the gripper is closed and $w_{TCP_{i},ring}$ exceed a threshold. The place SCT is implemented very similar to the pick SCT and makes use of the same VFs. It is visualized in Figure 5B, but not described in more detail here. After successfully picking a ring the follow_path SCT is activated as depicted in Figure 5C. The first state of the SCT is grasped. If *d* fulfills all criteria the transfer state is reached and a path following AF (PF) is set. If *d*_*x*_ indicates that $\Sigma_{TCP_{i}}$ is at the right side of the pad the at_target state is reached and the follow_path SCT finishes.

Each of the two benchmark scenarios starts with the pick SCT as the gripper is initially empty. The first benchmark progresses with the place SCT, while the second benchmark transitions to the follow_path SCT after grasping the ring successfully. As the distance between ***Σ***_*ring*_ and ***Σ***_*pin*_ becomes sufficiently small, the place SCT is activated as last task of both benchmark scenarios.

Six subjects performed both benchmark scenarios. They are split in two groups namely a test group and a control group. They have little to no experience with telemanipulation of the DLR MiroSurge System. Before starting with the benchmark scenarios a short introduction to the system is provided with a few introduction movements. The test group performed trials one and five of each benchmark scenario without assistance and the remaining trials with assistance. The control group performed each benchmark five times without assistance to investigate the hypothesis, that the proposed approach accelerates training. After each trial the participant is asked to rate the current workload based on the Overall Workload Scale (Vidulich and Tsang, 1987).

### 4.2 Results

To investigate the hypothesis that training with our approach enables a novice surgeon to realize a task faster, more accurately and with less cognitive load, the following metrics are presented: duration of a task Δ*t*, translational and rotational deviations from the predefined pose Δ***p***, consisting of Δ*p*_*trans*_ and Δ*p*_*rot*_, and the assessed workload. This section provides the pure results, a detailed interpretation of the results is provided in Section 4.3.

#### 4.2.1 Pose Deviation

The activated AFs attract $\Sigma_{TCP_{i}}$ towards the predefined pose by presenting a wrench ***w*** in each time step. We investigate Δ***p*** analyzing the recorded wrenches. For trials without assistance presented to the subjects, the VFs are calculated and used as evaluation metric but not haptically rendered to the input device and not affecting the robot’s movements.

**FIGURE 6:**
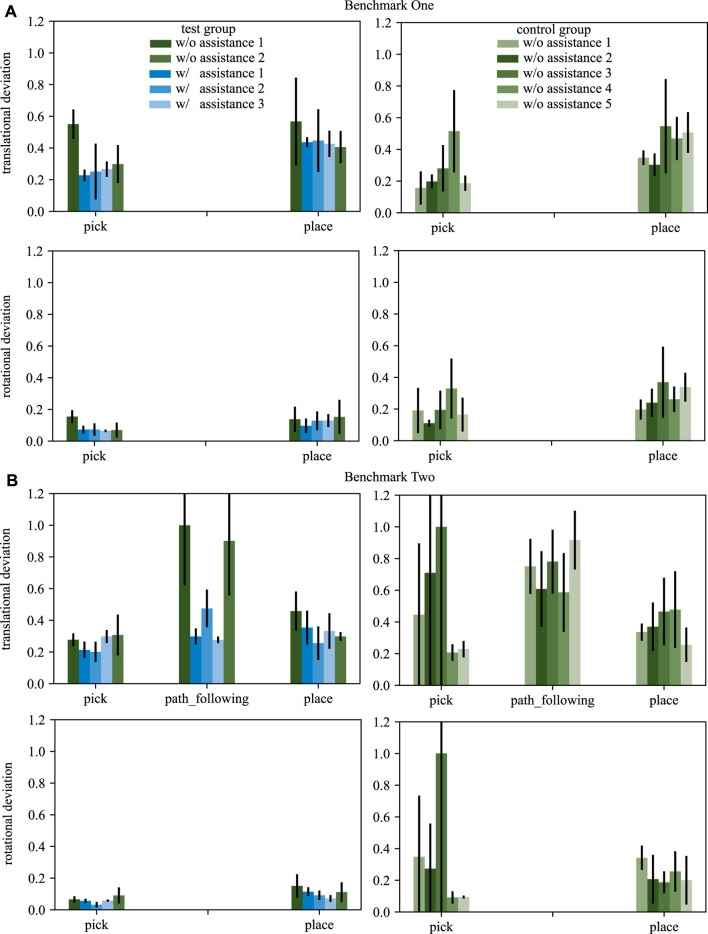
The translational and rotational deviations for benchmarks one **(A)** and two **(B)** are shown. The left column depicts results of the test group, the right column of the control group. Blue bars display trials with assistance, green ones trials without assistance. The standard deviation is depicted by black arrows.

The recorded wrenches are analyzed separately for each skill of the benchmark scenarios. Furthermore, the analysis splits wrenches into forces ***f*** and torques ***m***. The absolute force *f*_*t*_ measured in one time step is calculated from the forces along the x, y, *z*-axis by the euclidean norm. For each subject all *f*_*t*_ measured during one skill of one trial are accumulated to *f*_*a*_. *f*_*a*_ is normalized over all subjects and trials and over both benchmarks within one skill. Therefore, the results within one skill are comparable over both user groups and both benchmarks. Furthermore, the pick and place skills are normalized by the same value to make them comparable as well. The mean and standard deviation over all subjects’ normalized deviations is acquired separately for each of the trials. The recorded torques are processed equally. Figure 6 shows the processed deviations for both benchmark scenarios.

#### 4.2.2 Duration

**FIGURE 7:**
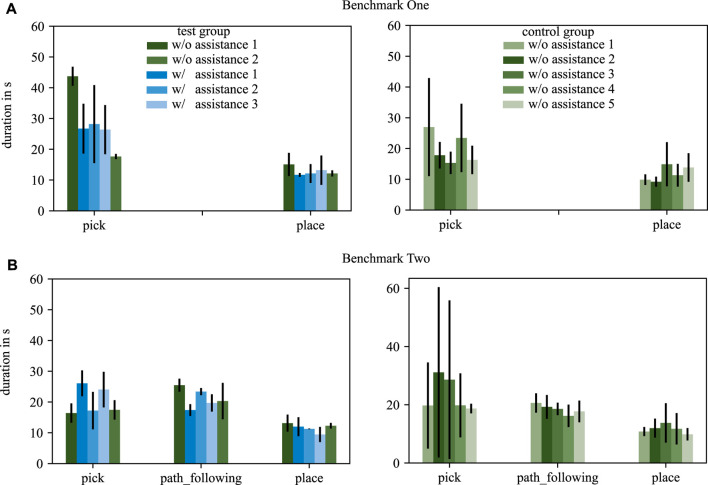
The duration of each tasks in benchmark scenario one **(A)** and benchmark scenario two **(B)** are depicted. The test group is displayed in the left column, the control group in the right column. Trials with assistance are shown in blue, trials without assistance in green. The standard deviation is depicted by black arrows.

The duration of completing a task is analyzed for each participant. Figure 7 shows the mean duration and standard deviation over all subjects for each trial for both benchmarks and both groups.

#### 4.2.3 Workload

**FIGURE 8:**
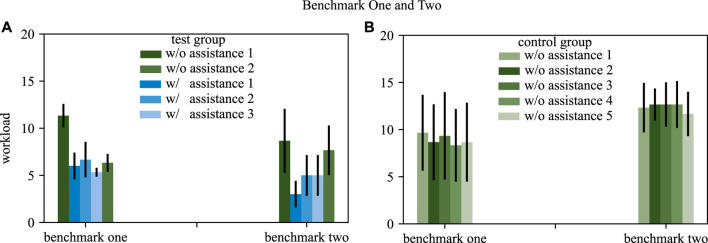
The workload for both benchmarks is depicted, comparing the test group **(A)** and the control group **(B)**. Blue bars depict trials with assistance, green bars trials without assistance. The standard deviation is shown by black arrows.

The workload is assessed after each trail with the Overall Workload Scale. The subjects are asked to rate their workload on a scale from 0 (very low) to 20 (very high) after each trial. The evaluation of the different tasks of a benchmark scenario is combined into one value. The mean and standard deviation is acquired by averaging over all participants but separating the different trials of a benchmark scenario. Figure 8 shows the mean and standard deviation over all subjects for each trial of both benchmark scenarios and both groups.

### 4.3 Performance Analysis

The small number of subjects included in this pilot study allows us to identify trends in how the proposed approach influences pose deviation, duration until benchmark scenario completion, and subjective workload. As each of the involved groups include only three participants the trends are presented in the form of plots rather then with a statistical analysis of the significance levels. The pilot study therefore does not aim to draw statistically significant conclusions, but instead to provide an initial assessment of feasibility.

#### 4.3.1 Pose Deviation

The analysis of the pose deviation aims to find trends investigating if our approach helps a novice surgeon to realize a task more accurately. The expected result is that after training with our approach the measured deviations of the predefined path are smaller than without such training represented by the control group. Figure 6 shows the processed deviations for both benchmark scenarios. The test group is depicted in the left column, the control group in the right.

In general, three main observations can be made. First, it is visible that the test group performs more accurate for some task if the assistance functions are activated (see trials with blue bars in Figure 6).

Second, the test group performs more accurate with respect to rotational deviation by means of absolute numbers, whereas the translational deviation is mostly comparable over all tasks. Third, comparing the performance (both translational and rotational) over all trials and all tasks of both benchmarks shows a clear trend: That is, for each task, the gradient on the mean deviation over the five trials is decreasing or constant for the test group, whereas it is increasing or constant for the control group.

Investigating the tasks individually, it is visible that especially the path_following SCT is providing assistance. For the trials in which the test group is assisted by means of haptic guidance, the accuracy is increased by 60%.

In addition, a learning effect can be observed for the test group, while the control group does not show a training effect. This training effect is further analyzed statistically by performing a two-sided related *t*-test comparing the first with the last trial for each task within each group. A two sided p-value *p* < 0.05 will be considered statistically significant. The resulting *p* reveal that the rotational deviation for the pick skill causes a statistically significant learning effect (*p* = 0.009). The remaining *p* are below the considered significance level. Considering that the statistical power of t-tests increases with the size of a data set, the result is deemed very promising and will be further investigated. The pick and place skills do not show a clear trend in the mean pose deviation. However, it is visible that the standard deviation is positively affected by the assistance functions for both tasks concerning translations and rotations.

In summary, the analysis of pose deviation shows that assistance functions have a certain impact on training for some tasks. Furthermore, the steady increase in pose deviation for some skills across both benchmarks for the control group shows that accuracy is further compromised as more repetitions are performed. This may already be an indication of increased workload, which will be discussed further at the end of this section.

#### 4.3.2 Duration

The duration of task execution is evaluated to investigate if SCOPE supports a novice surgeon to complete a required task faster. In comparison to the pose deviation, no general observation can be made with respect to this hypothesis.

Nevertheless, Figure 7 reveals that the test group improved the duration to complete the pick task, both with respect to mean values but as well with respect to the standard deviation. The place and path_following skills do not show clear effects in duration for both groups.

#### 4.3.3 Workload

The workload is assessed to investigate if SCOPE decreases the workload of a novice surgeon during task execution. The results shown in Figure 8 supports this hypothesis. In general, the analysis reveals that the perceived workload of the participants decreases when offering task dependent assistance functions. After training with our approach the execution of the required skills without assistance requires less workload then without training, which is also visible in detail when analyzing the two benchmark scenarios in detail.

Benchmark one shows that the workload decreases in the test group when repeating the same task multiple times. After some trials with assistance the workload for the trial without assistance is decreased compared to the first trial without assistance. The control group rates the workload for all trials in benchmark with a comparable value.

Benchmark two also shows that the trials with assistance for the test group are rated with less workload then the trials without assistance. The training effect is smaller comparing trial 1 and 5 without assistance. The control group rates do not show a learning effect in the workload for benchmark two.

## 5 Conclusion

This paper presents a novel approach to parameterize haptic assistance functions at run-time, entitled SCOPE. The procedural context to do so, is derived utilizing a DT approach. The concept is integrated into the DLR MiroSurge system and evaluated in a pilot study for surgical training. The experiments show a promising trend, that novel users profit from haptic augmentation during training of certain tasks, such that they perform a task faster, more accurately and with less cognitive load.

### 5.1 Scalability and Generalizability

Even though the pilot study is conducted with only a small amount of participants, it reveals promising trends with respect to the learning effect. In a next step, a follow-up user study will be conducted with partnering novice surgeons. This will allow the recording of a sufficiently large data set to analyze the statistical significance of the trends presented in this work.

As the portfolio of available skills in terms of SCTs is currently predefined by a technical expert, the amount of available skills is limited. The skill set will be extended by aligning the set of training tasks to established curricula (Ritter and Scott, 2007; Laubert et al., 2018; Chen et al., 2020), e.g., by introducing peg-transfer, cutting along resection lines, needle guidance and suturing. In addition, the available assistance functionality will be extended by parameterizable assistance functions such as motion scaling between input device and $\Sigma_{TCP_{i}}$.

The stiffness function parametrizing AF visualized in Figure 3 influences the surgeon’s haptic perception of the guidance. The preferred guidance mode might differ between surgeons and most likely influences training outcome. Therefore, we plan to automatically generate the stiffness function as extension to the empirically chosen function currently implemented for both benchmarks. Two approaches will be compared in the future improving different aspects of the haptic perception of VFs. First, the generation of the stiffness function will be based on trials performed by expert surgeons which describe the optimal stiffness. The second approach adapts the stiffness function on the surgeon’s preferences learned from previous trials.

The presented approach focuses on the task state estimation of a known task series. This approach could be extended by surgical gesture recognition inferring which task the surgeon aims to complete (van Amsterdam et al., 2021). This would allow for more flexible task series.

Furthermore it is crucial to note that the initial pose of all relevant objects in the scene is currently predefined. After initialization the objects’ poses are tracked by the physics simulation. The presented tasks consist of short sequences to prevent the divergence of the simulated scene from the real scene. Longer action sequences will require the integration of a vision pipeline to further prevent divergence of the simulated world and the real world. This includes object detection and tracking to allow for a more generic scene initialization and the update of the parameters, i.e., pose of the objects, used by the physics simulation. Additionally, the integration of a vision pipeline into our approach will enhance the flexibility of our setup.

### 5.2 Outlook

Based on the insights of this work, we argue that adaptable haptically augmented VFs explain a task more accurately than oral or visual explanations. In the future, the tasks to be trained could be learned from expert demonstrations (Steinmetz et al., 2019). To achieve this, an expert surgeon could perform a task first, allowing the abstraction of skills and corresponding support functions. A novice surgeon could then benefit from a haptically augmented training process that encodes the expert knowledge inherently.

As SCOPE allows the adaptable parametrization of VFs, it enables the personalization of VFs to the surgeons’ needs during the different phases of training. With increasing proficiency, the strength of the haptic guidance could be reduced to further train the novice surgeons’ ability follow the predefined path by their own (Enayati et al., 2018). According to Ovur et al. (2019) decreasing the strength of guidance and finally removing it doesn’t affect the achieved skill level. Furthermore, VFs could be parametrized according to the surgeon’s preferences observed by the robotic system.

At last, we envisage the possibility to train critical steps before surgery in virtual reality. Assistance functions could be predefined in virtual reality and applied during real surgery. However, the automatic perception of procedural context information in real surgery is still an open research question. Perceiving the procedural context would allow to integrate SCOPE into real surgical procedures.

This outlook leads to the final goal of supporting surgeons during MIRS with individualized contextual assistance and therefore enhancing surgical performance.

## Data Availability

The datasets presented in this study can be found using the following link: https://github.com/DLR-RM/Contextual-MIRS-Assistance.

## Footnotes

1 For more details on the control of the system, please refer to Le Tien et al. (2007) and Le Tien et al. (2008) for the MIRO, to Thielmann et al. (2010) for the MICA drive unit and to Tobergte et al. (2009) and Seibold et al. (2018) for the overall system control.
